# Ultrasound-enhanced ocular delivery of dexamethasone sodium phosphate: an *in vivo* study

**DOI:** 10.1186/2050-5736-2-6

**Published:** 2014-03-31

**Authors:** Marjan Nabili, Aditi Shenoy, Shawn Chawla, Sankaranarayana Mahesh, Ji Liu, Craig Geist, Vesna Zderic

**Affiliations:** 1 Department of Electrical and Computer Engineering, George Washington University, Washington, DC 20052, USA; 2 Department of Ophthalmology, George Washington University, Washington, DC 20052, USA

**Keywords:** Therapeutic ultrasound, Drug delivery, Cornea, Ocular diseases, Sonophoresis, Dexamethasone sodium phosphate

## Abstract

**Background:**

The eye's unique anatomy and its physiological and anatomical barriers can limit effective drug delivery into the eye.

**Methods:**

An *in vivo* study was designed to determine the effectiveness and safety of ultrasound application in enhancing drug delivery in a rabbit model. Permeability of a steroid ophthalmic drug, dexamethasone sodium phosphate, was investigated in ultrasound- and sham-treated cases. For this study, an eye cup filled with dexamethasone sodium phosphate was placed on the cornea. Ultrasound was applied at intensity of 0.8 W/cm^2^ and frequency of 400 or 600 kHz for 5 min. The drug concentration in aqueous humor samples, collected 90 min after the treatment, was determined using chromatography methods. Light microscopy observations were done to determine the structural changes in the cornea as a result of ultrasound application.

**Results:**

An increase in drug concentration in aqueous humor samples of 2.8 times (*p* < 0.05) with ultrasound application at 400 kHz and 2.4 times (*p* < 0.01) with ultrasound application at 600 kHz was observed as compared to sham-treated samples. Histological analysis showed that the structural changes in the corneas exposed to ultrasound predominantly consisted of minor epithelial disorganization.

**Conclusions:**

Ultrasound application enhanced the delivery of an anti-inflammatory ocular drug, dexamethasone sodium phosphate, through the cornea *in vivo*. Ultrasound-enhanced ocular drug delivery appears to be a promising area of research with a potential future application in a clinical setting.

## Background

The objective of this study was to investigate the role of ultrasound in enhancing delivery of an ocular anti-inflammatory drug, dexamethasone sodium phosphate, through the cornea in a rabbit eye model *in vivo*. Ultrasound has been used in ophthalmology for decades but mostly as a diagnostic imaging tool [1]. For example, ultrasound biomicroscopy imaging can provide high-resolution imaging of the anterior part of the eye [2]. Therapeutic ultrasound also has a potential for clinical applications in ophthalmology [3]. High-intensity focused ultrasound (HIFU) has been reported as an effective treatment method for reducing intraocular pressure, which showed promising results in both animal and clinical studies as a potential novel approach for glaucoma treatment [4-6]. Sonoda et al. [7] used ultrasound in conjunction with commercially available microbubbles to enhance gene delivery into the back of the eye of New Zealand albino rabbits without any ocular tissue damage observed and with 1.5 to 2 times increase in the delivery efficiency. The application of therapeutic ultrasound at frequency of 690 kHz has also been observed as effective in moderately disrupting the integrity of the blood retinal barrier when applied as 10 ms bursts of 1 Hz for 60 s at pressures of up to 1.1 MPa, thus increasing the penetration of systemically administered drugs into the retina [8].

The eye's distinct anatomy and physiology pose challenges to the development of effective ocular drug delivery systems [9] since its various defense mechanisms make it very difficult to achieve sufficient drug penetration into the eye [3,10]. Topical administration of drugs into the eye is convenient, common, and well accepted by patients [9,11]. However, this method is highly inefficient because only 1%–5% of the applied ocular drug penetrate to the desired ocular site [11,12]. Most of the topically administrated drugs are washed away by factors such as normal tear volume, blinking, induced lacrimation [12], and naso-lachrymal drainage [9,11,12]. Moreover, drugs can enter systemic circulation through conjunctival blood capillaries and lymphatics before reaching intraocular tissues [12,13]. The cornea is considered to be the main pathway for topical drug delivery [3,10]; however, the passage of ocular drugs is limited by corneal barriers [9-11]. This highly selective barrier consists of three primary layers: namely, the epithelium, which is impermeable to hydrophilic drugs because of its lipophilic properties and tight junctions; the stroma, which is hydrophilic in nature, making it a dominant barrier for lipophilic drugs; and the endothelium [14,15]. Intravitreal and periocular injections are some of the invasive methods of ocular drug delivery, which are used to avoid inefficient topical and systemic processes. However, these methods have their own side effects including infection and cataract formation [3]. Many other methods such as hydrogel contact lenses, muco-adhesive and viscosity-enhancing polymers, iontophoresis, liposomes, and nanoparticles have been used to increase concentration of the drugs delivered into the eye [12,13]. For example, hydrogel contact lenses can increase the bioavailability of drugs by prolonging the drug exposure time. However, these contact lenses can cause blurring of vision and local corneal tissue toxicity [16,17]. Ocular iontophoresis, while effective in delivering drugs, requires specialized drug formulations and was shown to have mild adverse effects [18]. This noninvasive technique involves applying a small electric current to enhance the penetration of ionized drug into the eye [9] and has been investigated for delivering different types of ocular drugs such as antibiotics [19] and anti-viral drugs [20]. Injectable biodegradable implants in the forms of rods, plugs, discs, or sheets have also been proposed for ocular drug delivery; however, this approach is expected to have low patient compliance due to its invasive nature [21].

The design of our study focused on promoting topical drug penetration through the cornea via ultrasound methods, potentially providing a way for topical ophthalmic drugs to reach different areas in the eye more effectively. Our approach may allow for better targeting of diseased eye tissues as compared to systemic methods, leading to usage of smaller quantities of the drug to get the desired therapeutic effect and to reduce toxicity of the treatment. In addition to delivery of commercially available ophthalmic drugs, other compounds such as macromolecules that may assist with wound repair have the potential to be coupled with ultrasound-mediated delivery [22]. Wound repair in the body is normally dependent on the presence of vasculature for the delivery of nutrients to damaged tissue; however, many of the eye's translucent structures are avascular (e.g., cornea, lens, vitreous). When damaged, these tissues do not have access to thrombospondin 1, a glycoprotein central to tissue repair, and as a result fibrosis and repair are slow, causing scarring of eye tissue and thus blindness [23].

Results from our previously performed *in vivo* studies showed that using 880-kHz ultrasound at intensities of 0.19, 0.34, and 0.56 W/cm^2^ (in a continues mode) with an exposure duration of 5 min resulted in 2.4, 3.8, and 10.6 times increases in corneal permeability respectively for a drug mimicking dye sodium fluorescein [24]. Similar results were also reported by Nuritdinov [25] who showed that ultrasound at frequencies of 470–880 kHz and intensity of 0.3 W/cm^2^ applied for 5 min produced up to ten times increase in the corneal permeability for sodium fluorescein in a rabbit model, *in vivo*.

The main goal in the study reported here was to investigate the corneal permeability for a clinically relevant anti-inflammatory drug, dexamethasone sodium phosphate, in an *in vivo* model, which can give us a better understating of an actual clinical situation. After investigating the effects of ultrasound application on the delivery of dexamethasone sodium phosphate *in vitro*[26], we decided to focus on the ultrasound parameters found optimal in these *in vitro* studies and utilize them in our *in vivo* studies.

## Methods

A total of 20 healthy New Zealand white rabbits, 1–2 years old, were used in our studies. Rabbit eyes have been used as the standard model for ophthalmic drug delivery research [14,27,28]. All procedures were performed in accordance with Institutional Animal Care and Use Committee (IACUC) regulations under the approved protocol. The entire experiment was completed in the animal facility at the George Washington University in the span of two nonconsecutive days. An attending veterinarian assisted our research team with anesthesia and animal handling procedures. Rabbits were anesthetized with injection of ketamine (50 mg/kg) and xylazine (5 mg/kg). Anesthesia was reapplied as needed as determined by the veterinarian.

### *In vivo* experiment

The rabbits were randomly assigned to three groups: ultrasound treatment at 400 kHz (*n* = 6), ultrasound treatment at 600 kHz (*n* = 6), and sham treatment group (*n* = 8). The sham treatment experiments were set up exactly the same as ultrasound-treated experiments. Ultrasound transducer was submerged inside the solution in eye cup, and all of the procedures were the same (except that the ultrasound power button was not turned on). In all cases, only one of the rabbit's eyes received the treatment. The other eye was used as a control where no ultrasound application or drug exposure was present. These control eyes were used for histological observations. The ophthalmic drug used in our experiments was dexamethasone sodium phosphate, which had also been used during our previous *in vitro* studies [26]. Dexamethasone sodium phosphate is used in ophthalmology as a 0.1% topical steroid solution (Bausch & Lomb Inc, Tampa, FL, USA) applied in the treatment of inflammatory conditions. It has a molecular weight of 516.41 Da and hydrophilic properties.

Unfocused circular ultrasound transducers (Sonic Concepts, Bothell, WA, USA) with 15-mm active diameters were used in the experiments and were positioned at near to far field transition distance from the eye to ensure optimal energy delivery. The transition point from the near field to the far field, *d*_ff_, is the location of the furthest maximum pressure for the unfocused transducer [29]. These transducers operated at frequencies of 400 and 600 kHz, and the *d*_ff_ calculated for each transducer frequency was 1.5 and 2.25 cm, respectively. The driving unit consisted of a function generator (33250, Agilent, Santa Clara, CA, USA) connected to the power amplifier (150A100B, Amplifier Research, Souderton, PA, USA), which was connected to the ultrasound transducer via an electrical power meter (Sonic Concepts, Bothell, WA, USA).

**Figure 1:**
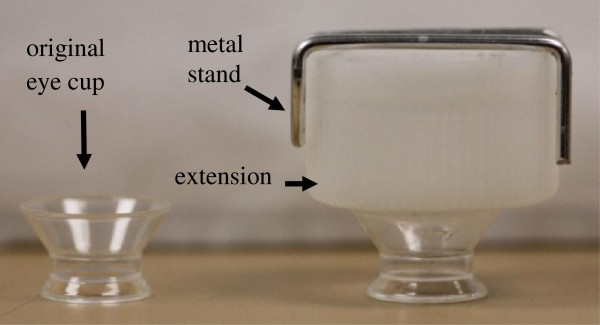
**Eye cup design.** Original and modified eye cup used in *in vivo* experiments.

The eye cup was designed in such a way that it could hold up to 25 mL of solution by adding an extension above the standard eye cup area as shown in Figure 1. This extension was added to ensure that the transducers would be submerged in the drug solution while still held within the *d*_ff_ distance from the corneal surface. A metal xstand was placed inside the eye cup to secure the transducers in place and keep them at *d*_ff_ distance from surface of the cornea during ultrasound and sham treatments. Ultrasound transducer was submerged in the drug solution inside the eye cup during the experiment.

**Figure 2:**
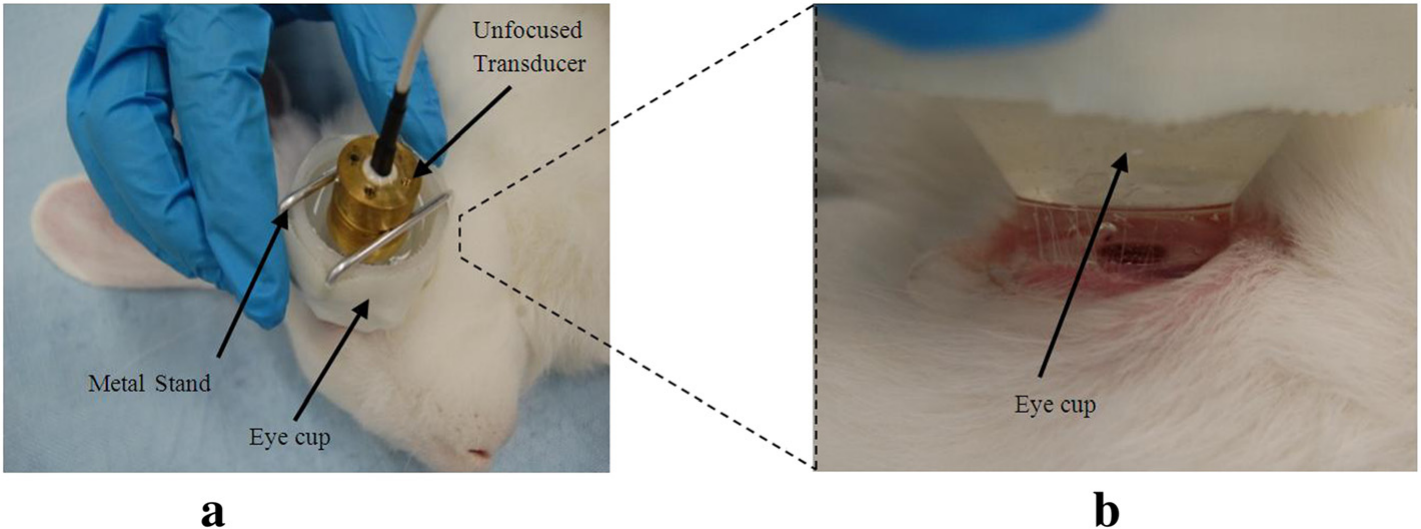
***In vivo *setup. (a)** The eye cup and transducer placement. **(b)** Close-up of the eye cup placement on the rabbit eye *in vivo*.

During treatment, therapeutic ultrasound was applied at the parameters that were shown most effective in our previous *in vitro* studies. The *in vitro* results specifically showed that using lower frequency ultrasound (in the 400 kHz to 1 MHz range) was more effective in increasing the permeability of the cornea [26]. The ultrasound intensity used in the *in vivo* studies was 0.8 W/cm^2^, and duration of ultrasound application was 5 min. The ultrasound transducers and the eye cup were rinsed with saline before procedure. The eye cup (Figure 2) was placed under the rabbit eyelid and filled with the drug solution after the animal was anesthetized. Ultrasound application started immediately after the eye cup was placed on the eye and filled with the drug solution. The transducer with the active diameter of 15 mm covered the entire surface of the cornea which had a diameter of 14–15 mm. After 5 min, ultrasound application was stopped and the transducer and the drug solution were removed from the eye cup, followed by a careful removal of the eye cup from the animal. The eye was not rinsed afterwards.

**Figure 3:**
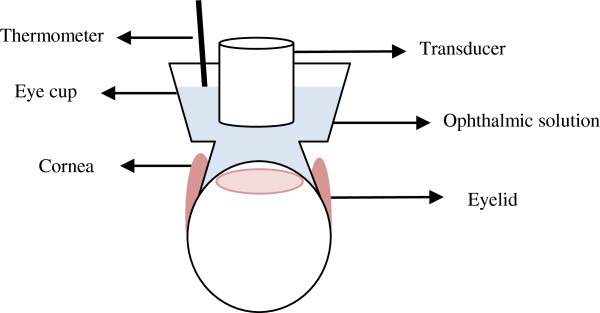
**Schematic of *in vivo *setup.** Placement of the thermocouple and transducer with respect to the eye.

The solution was kept at room temperature (approximately 25°C) before the starting of the experiments. The temperature of drug solution inside the eye cup in the proximity of the cornea was measured (Figure 3) during ultrasound application using a thermometer (dual thermometer, Fisher Scientific, Atlanta, GA, USA). The thermocouple used was a K type with approximately 1-mm width. The thermometer was not touching the cornea to prevent accidental damage. Temperature was recorded at initial (*t* = 0 min), midpoint (*t* = 2.5 min), and final (*t* = 5 min) times after the start of ultrasound application.

After ultrasound application, *in vivo* gross observation of the cornea was performed using a high-magnification stereomicroscope. This procedure was repeated after 80 min from the start of the experiment, before the animal was euthanized.

At approximately 90 min after the ultrasound treatment, the animal was euthanized (using Euthasol at 1 mL/4.54 kg) and 0.3 mL sample of the aqueous humor was collected subsequently using a 27 G × 1/2 in. needle (12.7 mm length). The aqueous humor samples were stored in a freezer at −80°C. Once the aqueous humor was sampled, the eyes were excised and the cornea was dissected and fixed in formalin (Protocol®, Fisher Scientific Company, Kalamazoo, MI, USA).

### Histology

The cornea samples were prepared using hematoxylin and eosin (H & E) staining procedures for histology slides (Histoserv, Inc., Germantown, MD, USA). In our previous *in vitro* studies [26], we developed a procedure for the determination of changes in the epithelial layer of the cornea, where corneas were classified into four groups based on the level of epithelial damage. Each class was given a value of 0, 1/3, 2/3, or 1 (please see Figure 2 from Nabili et al. [26]). Class 0 (value of 0) has no epithelial disorganization (all cells appear intact; the layers of epithelium are well organized). Class 1 (value of 1/3) has minor epithelial disorganization (some of the cells appear necrotic; some cells are missing or the first layer of epithelium is removed). For class 2 (value of 2/3), a more severe epithelial disorganization is observed (more cells appear necrotic or missing; cells in the two to three layers of epithelium appear damaged). Class 3 (value of 1) has the most severe epithelial disorganization (majority of the cells are ruptured; all of the epithelial layers are absent or severely damaged).

The thickness of the different layers of the cornea (epithelium, stroma, and endothelium) was also determined from the histological slides. To determine the thickness, measurements of the width of each layer, were performed at the same three locations on each sample. Each cornea sample was examined using Zeiss AxioImager light microscope (Carl Zeiss, Jena, Germany) at × 5–20 magnification. The three locations that were measured on each cornea sample were at the center of the cornea (location 1), midway between the left end of the cornea and the center (location 2), and midway between the right end of the cornea and the center (location 3). These measurements were reported in micron. If any of the corneal layers were missing, they were recorded as ‘absent’ and were shown in calculations as a ‘0’ value.

### Chromatography

Aqueous humor samples were sent to a facility (Cayman Chemical Co., Ann Arbor, MI, USA), which used chromatography methods to investigate the amount of dexamethasone sodium phosphate inside sham- and ultrasound-treated samples. A calibration curve for dexamethasone sodium phosphate was first generated, followed by the determination of the amount of dexamethasone sodium phosphate in aqueous humor samples. Two out of twenty samples sent to the facility were not analyzed due to inadequate amounts of aqueous humor in these samples.

Based on the protocol provided by this chromatography facility, the procedure to generate calibration curves for dexamethasone and measuring amount of dexamethasone in an aqueous humor sample is explained as follows: to create the calibration curve for dexamethasone, 10 μL of dexamethsone calibration standard solution (100 μg/mL) was added to a microcentrifuge tube with 990 μL of 95:5 (*v*/*v*) water/acetonitrile, vortex mixed for about 15 s, and subsequent 500 μL serial dilutions were made. Fifty microliter of dexamethasone-d4 internal standard solution was added to 100 μL of each standard and vortexed for approximately 15 s; 1 mL of methyl *tert*-butyl ether (MTBE) was added to each and vortexed for about 15 s and then centrifuged for 10 min. The organic layer was transferred to a 1.5-mL auto-sampler vial and dried under nitrogen and reconstituted with 100 μL of 95:5 (*v*/*v*) water/acetonitrile. To investigate the amount of dexamethasone inside the aqueous humor sample from our experiments, 10 μL of sample with unknown concentration of dexamethasone sodium phosphate and 90 μL of water were added to a microcentrifuge tube, and 50 μL of dexamethasone-d4 internal standard solution was added to one 100 μL of each standard and vortexed for about 15 s. One milliliter of MTBE was added to 46 each, vortexed about 15 s, and centrifuged for 10 min. The organic layer was transferred to a 1.5-mL auto-sampler vial and dried under nitrogen and reconstituted with 100 μL of 95:5 (v/v) water/acetonitrile.

## Results

**Figure 4:**
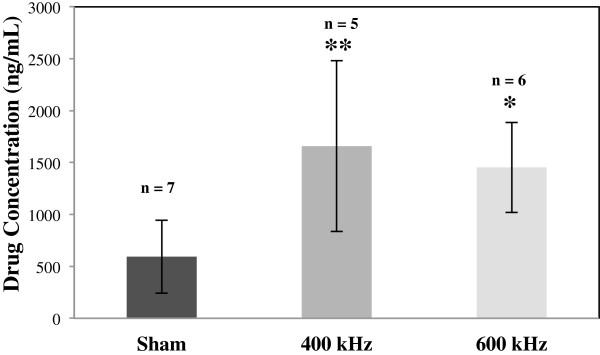
**Dexamethasone sodium phosphate concentration in aqueous humor.** Dexamethasone sodium phosphate concentration increased in aqueous humor as a result of ultrasound application as compared to sham-treated samples (*n* = 7). Ultrasound was applied at frequencies of 400 kHz (*n* = 5) and 600 kHz (*n* = 6), intensity of 0.8 W/cm^2^, and exposure duration of 5 min. Data are given as mean ± standard deviation. *p value <0.05, **p value <0.01.

The statistical test used to analyze the data was a *t*-test: two-sample assuming unequal variances. Figure 4 shows the comparison between the dexamethasone sodium phosphate concentration in aqueous humor in ultrasound-treated and sham-treated samples. The increase in drug concentration in aqueous humor samples was 2.8 times (*p* < 0.05) at ultrasound frequency of 400 kHz (*n* = 5) and 2.4 times (*p* < 0.01) at frequency of 600 kHz (*n* = 6), when compared to sham-treated samples (*n* = 7). Drug concentration increase in the eye in case of sham-treated samples was 593.8 ± 351.9 ng/mL. This value was 1,658.5 ± 823 ng/mL using 400-kHz frequency and was 1,453.8 ± 434.3 ng/mL using 600-kHz-frequency ultrasound.

**Figure 5:**
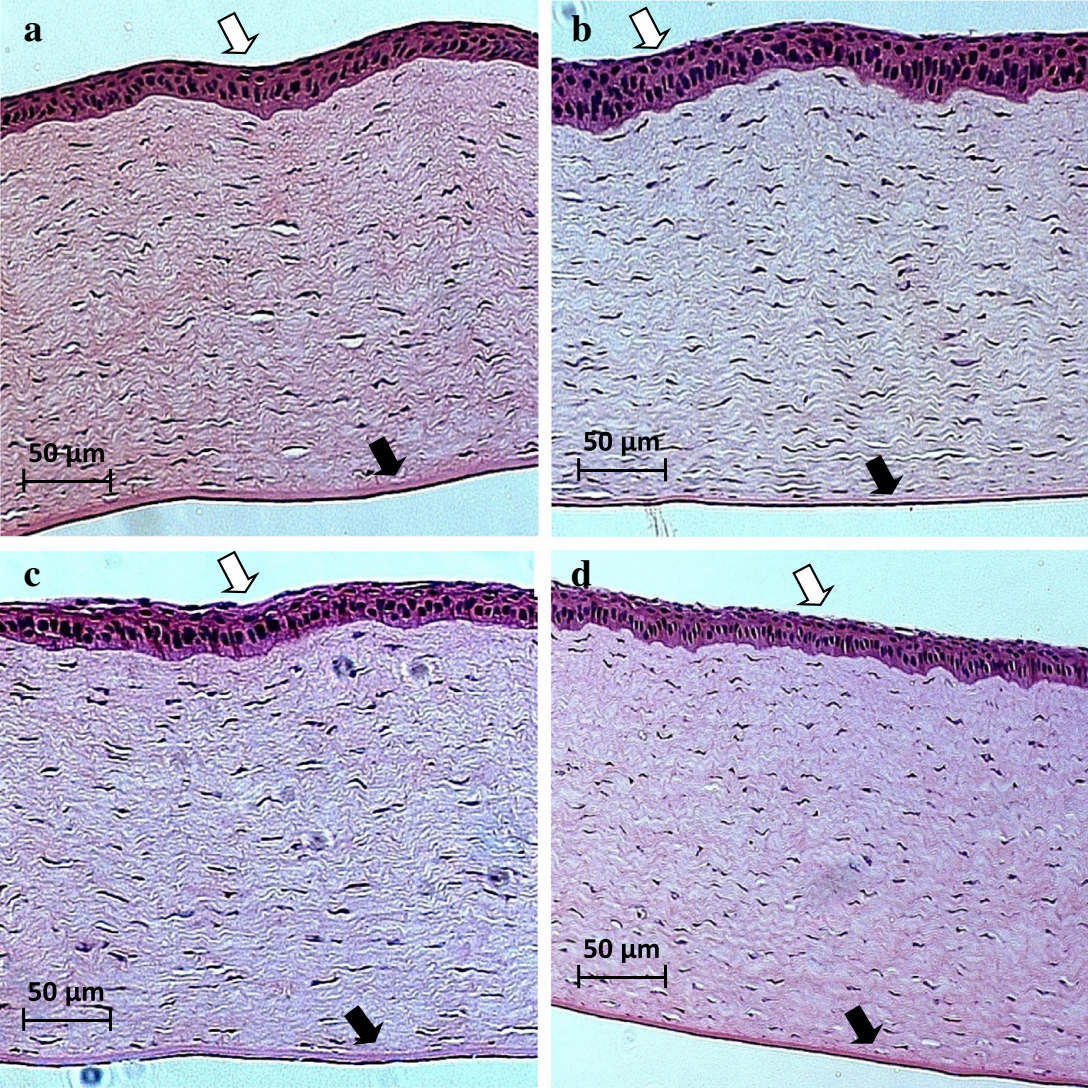
**Histology analysis. (b)** A control cornea, **(b)** a sham-treated cornea, **(c)** a corneal sample exposed to 400-kHz ultrasound, and **(d)** a corneal sample exposed to 600-kHz ultrasound. The epithelium is the eosinophilic layer, indicated by *white arrow*, as opposed to much thinner endothelium, which is just a cell layer in thickness, marked with a *black arrow*. Magnification of × 10 was used.

**Figure 6:**
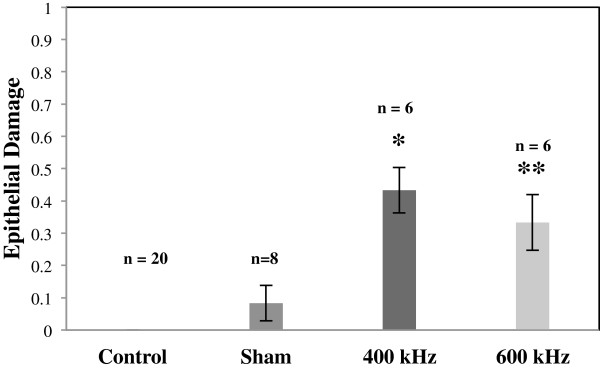
**Change in the corneal structure.** Changes in the cornea as a result of ultrasound application as compared to sham-treated corneas (*n* = 8). Different *bars* represent the corneal damage due to ultrasound application at frequencies of 400 kHz (*n* = 6) and 600 kHz (*n* = 6). Data are shown as mean ± standard deviation. No changes were observed in control corneas (*n* = 20) in all cases. *p value <0.05; **p value <0.001.

No gross damage of the cornea was detected using stereomicroscopy observations immediately after the treatment and 80 min after the treatment. In subsequent histological observations, ultrasound-induced changes were observed in the epithelial layer of the cornea including missing cells and in some cases detachment of whole epithelial cell layers. No apparent changes were observed in the stroma and endothelium; however, detachment of the endothelium was observed in some sham-treated and ultrasound-treated samples and may be caused by processing artifacts [30,31]. Representative light microscopy images of control, sham-treated, and ultrasound-treated corneas are shown in Figure 5. Results of histological analysis of ultrasound- and sham-treated corneas based on the four classes of epithelial damage, as described in the ‘Methods’ section, are shown in Figure 6. The increase in the epithelial damage was four times (*p* < 0.01) at ultrasound frequency of 400 kHz (*n* = 6) and three times (*p* < 0.05) at frequency of 600 kHz (*n* = 6), as compared to sham-treated samples (*n* = 8).

The quantitative changes in the corneal structure was 0.25 ± 0.46 in sham-treated samples, 1.7 ± 0.51 using 400-kHz frequency, and 1.0 ± 0.63 using 600-kHz-frequency ultrasound. These values are calculated based on the characteristic model designed to analyze corneal damage in semi-quantitative matter (see ‘Methods’ section).

Despite the fact that aqueous humor samples from all eyes were sent to a chromatography facility, the amount of drug in one sample from 400-kHz batch and one from sham-treated batch was not investigated due to inadequate amount of samples for the equipment. However, all cornea samples which were sent to a histological facility were processed. For these reasons, there is a difference between samples of aqueous humor and cornea samples used for histology analysis.

**Figure 7:**
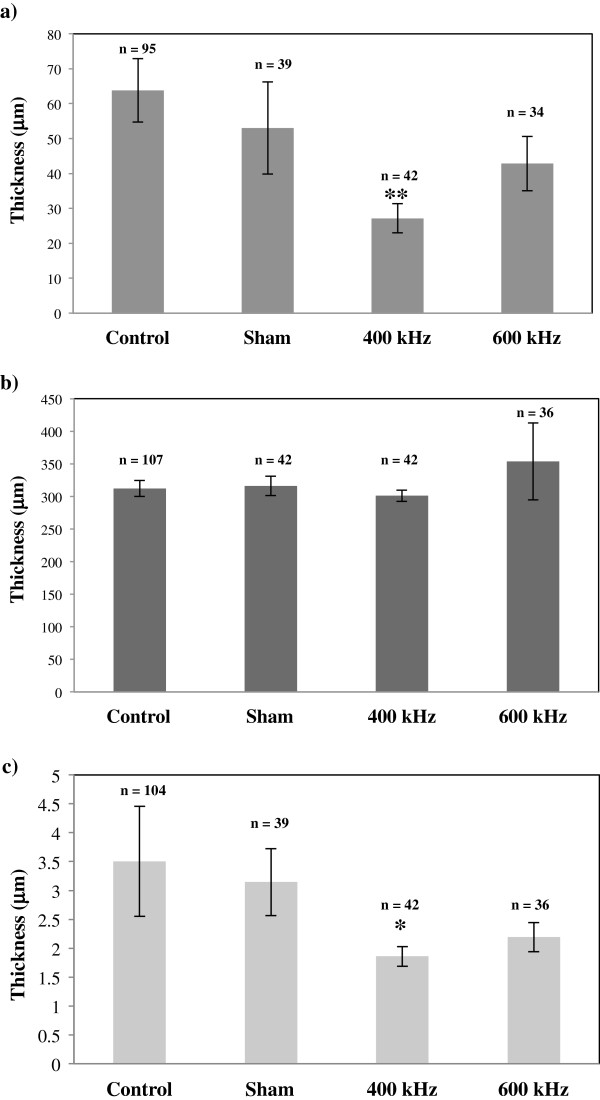
**Thickness of different layers of the cornea.** This graph shows thickness of the different layers of the cornea: **(a)** epithelium, **(b)** stroma, and **(c)** endothelium. Different *bars* represent results for control, sham-treated, 400- and 600-kHz-ultrasound-treated samples. *p value <0.05 as compared to sham values; **p value <0.001 as compared to control values.

Figure 7 shows thickness of different layers of cornea in ultrasound- and sham-treated samples. The mean thickness of ultrasound-treated epithelium was smaller as compared to control and sham-treated cases. For 400-kHz ultrasound exposure, changes in the epithelial thickness were observed as 57% (*p* < 0.001) decrease as compared to control epithelium and 49% decrease as compared to sham-treated samples (Figure 7a). For 600-kHz ultrasound treatments, apparent decrease in the epithelial thickness was 33% as compared to control samples and 19% as compared to sham-treated samples (with no statistical significance) (Figure 7a). The thickness of the ultrasound-treated stroma was not significantly different from control and sham-treated stroma (Figure 7b). Endothelial thickness appeared to decrease to 10% for sham-treated samples as compared to controls with no statistical significance. Comparison between sham-treated and ultrasound-treated endothelium showed 41% decrease (*p* < 0.05) and 30% decrease for 400 and 600 kHz, respectively (Figure 7c).

**Figure 8:**
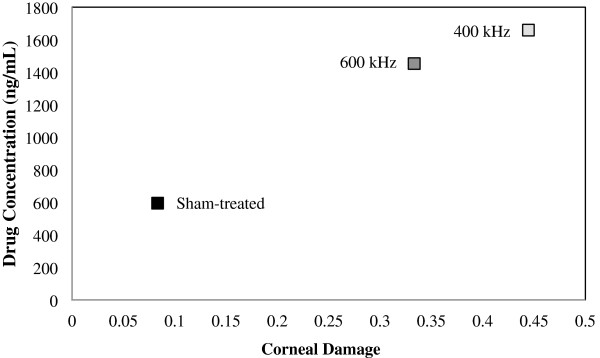
**Relation between drug concentration in aqueous humor and corneal damage.** This figure shows a relationship between corneal damage and drug concentration in the aqueous humor for ultrasound- and sham-treated cases. Data are given as mean ± standard deviation.

Figure 8 shows the relation between drug concentration in the aqueous humor and the corneal damage as calculated based on the four classes of epithelial damage (as shown in Figure 6). There appears to be a direct relation between the drug concentration increase and the level of corneal damage.

The change in temperature from *t* = 0 to *t* = 5 min for sham-treated cases was 0°C–3°C (mean ± standard deviation of 1.4°C ± 1.2°C). In ultrasound-treated cases, the change of temperature for the same period of time was 3°C–6°C (mean ± standard deviation of 4.0°C ± 1.1°C) for 400-kHz ultrasound and 4°C–5°C (mean ± standard deviation of 4.8°C ± 0.4°C) for 600-kHz ultrasound.

## Discussion

The potential advantages of using ultrasound for drug, gene, and protein delivery, using parameters that cause cavitation and streaming effects, have been confirmed in different experimental studies [24,28,32]. Cavitation is known as the creation, oscillation, and collapse of gas bubbles due to acoustic waves [33-35]. It is stated to be the main mechanism responsible for promoting drug delivery through the skin [32,36]. There are two types of cavitation: stable and inertial. Stable cavitation is defined as the uniform pulsation of bubbles over long time intervals [33], and the oscillation of these bubbles can produce mechanical stresses that may cause cell membrane rupture [37]. In the case of inertial cavitation, collapse of the bubbles causes shock waves and microjets to be generated near the cells, thus producing pits in the cell membranes [38,39]. The effect of inertial cavitation is greater at lower frequencies because bubbles have more time to grow, which results in a more violent collapse [40]. Based on our previous studies [24], at ultrasound frequency of 880 kHz, cavitation activity was shown to correlate with enhancement of drug delivery through the cornea *in vivo*, and similar mechanisms are expected here. The mechanism, which has an effect on increasing the drug penetration through the cornea, is thought to be cavitation and generation of bubbles. In this study, stable cavitation appeared to be the cause of corneal permeability enhancement in the case of ultrasound applied at 0.19 W/cm^2^, however; both stable and inertial cavitation were present when higher intensities of 0.34–0.56 W/cm^2^ were used [24].

There is an inverse relationship between ultrasound frequency and cavitation effects. It is difficult to generate cavitation at higher frequency because the time between the positive and negative acoustic pressure is short [41]. As a result, the gas that is dissolved in a medium does not have the ability to diffuse into the cavitation nuclei [41]. For the purpose of this study, we chose ultrasound frequencies of 400 and 600 kHz which belong to medium frequency range; therefore, cavitation was one of the likely mechanisms that cause an increase in corneal permeability. Results from our *in vitro* study for dexamethasone sodium phosphate showed that corneal permeability had a statistically significant increase of 43% to 109% after ultrasound exposure at 400 kHz (at intensity of 0.3 to 1.0 W/cm^2^ applied for 5 min) as compared to sham-treated samples. The increase in corneal permeability observed in case of 400-kHz ultrasound was more effective as compared to ultrasound application at 600 kHz (46%–55% permeability increase at intensities of 0.3 to 1.0 W/cm^2^), 800 kHz (50%–72% increase at intensities of 0.5 to 1.0 W/cm^2^), and 1 MHz (46%–63% increase at intensities of 0.5 and 0.8 W/cm^2^).

In addition to cavitation effects, streaming and microstreaming may have played a role in increasing corneal permeability. Acoustic streaming is identified as a force, which is able to move ions and small molecules as a result of physical action of ultrasound waves [42]. Shearing and streaming forces associated with ultrasound may disrupt cell walls, internal organelles, and tissues if applied at high enough intensities [29]. Further, acoustic streaming was recognized to be an important factor in the enhancement of drug delivery through the skin [32,36]. When bubbles oscillate in an ultrasound field, a microscale circulatory motion forms around the oscillating bubble and is called microstreaming [43,44]. The shear stresses generated by microstreaming near cell boundaries, due to expansion and contraction of microbubbles, may cause cell membrane rupture [37] and result in hemolysis, release of protein from bacteria, and mechanical disruption of plant cells [37,45]. Further, microstreaming was shown to cause an increase in the movement of blood and fluids around the thrombus and enhance drug infusion into the thrombus [46]. Therefore, streaming and microstreaming are important ultrasound mechanisms that have the potential to increase drug penetration through the cornea in this study.

The epithelium, stroma, and endothelium of the cornea each have its own unique structure, which can represent a barrier for drug penetration into the eye [47]. The corneal epithelium's tight junctions and its lipophilic nature act as a barrier to hydrophilic drugs, and the stroma's highly hydrated structure limits the penetration of lipophilic drugs [47]. Any corneal disorganization caused by ultrasound application, which changes the structure of the first layer of the cornea and its tight junctions, is expected to enhance the drug penetration into the eye [26,48]. In comparison, drug penetration through the skin also increased as ultrasound application altered the stratum corneum and its lipid bilayer structure [32].

The *in vivo* study presented here is designed based on our previously performed *in vitro* study, which resulted in a statistically significant increase in the corneal permeability for dexamethasone sodium phosphate [26]. Results from our *in vitro* study showed a corneal permeability increase of 2.1 times at 400 kHz and 1.5 times at 600 kHz, at intensity of 0.8 W/cm^2^, as compared to sham-treated samples. Likewise, this *in vivo* study showed a statistically significant increase in drug delivery through the cornea. The increase in corneal permeability was greater in our *in vivo* study with maximum increase of 2.8 times, as compared with previous *in vitro* experiments [26]. In both studies, the level of damage observed in histology slides was limited to the epithelial layer, with no apparent damage in the stroma and endothelium [26].

The quantity of drug used was adequate to insure submerging of ultrasound transducer tip inside the solution. The maximum amount of drug used in the eye cup was approximately 15 mL. There is no direct relationship between the quantity of drug used in our study and the quantity of drug delivered locally in eye drops. In our study, the eye was exposed to drug solution for 5 min, which is less than the lag time (approximately 25 min) allowing penetration of dexamethasone sodium phosphate through the cornea. The goal of this study was not only to compare the eye drops and the eye cup drug application but also to see if ultrasound can promote the drug delivery through the cornea *in vivo*.

In our previous *in vitro* studies, the spherical head diffusion cell was used to minimize the change to the natural shape of the eye, but this change in the eye structure as compared to *in vivo* situation may have been a contributing factor—likely minor to the enhancement of corneal permeability. Also, storing the eyes in nutrient-adjusted storage solution after the eyes are excised can cause deterioration of corneal barrier properties [49] which may increase corneal permeability *in vitro* in both ultrasound-treated and sham-treated samples. In summary, the advantages of testing *in vivo* as compared to *in vitro* setup are the following: keeping the natural shape and structure of the rabbit eye, natural clearance mechanisms, perfusion, and the fact that eye does not potentially lose its barrier properties due to decay [49,50].

Investigating the corneal permeability of dexamethasone sodium phosphate, using 400- and 600-kHz ultrasound frequencies at 0.8 W/cm^2^ for 5 min, resulted in the enhancement of ocular drug delivery through the cornea, which was statistically significant as compared to sham-treated samples in *in vitro* and *in vivo* studies. There are differences in results from *in vitro* and *in vivo* studies that may be caused by experimental setup, environmental aspects, and tissue orientation. For example, the absorption of samples collected from receiver compartment of diffusion cells during *in vitro* study was measured using a spectrophotometer. The sensitivity of this device can have an impact on the amount of drug detected. On the other hand, sampling the actual aqueous humor from the eye in *in vivo* study and measuring the drug concentration inside this solution using chromatography methods can be more efficient. Moreover, all the samples in both cases were treated the same and were analyzed consistently.

In our current study, the major structural changes were observed in the epithelial layer of the cornea. Some of these changes included cell removal from the first layer of the epithelium. Also some epithelial cells from inner layers appeared lighter in color or their nucleus were missing. The stroma and endothelium were not damaged in most cases; however, detachment of endothelium was observed in some samples, which may be caused by processing artifacts [30,31]. Our current findings were consistent with our previous work where based on histological analysis, in both *in vitro* and *in vivo* corneal samples, the ultrasound-induced changes were observed mostly in the epithelial layers [24,26,48]. Results from our semi-quantitative analysis (as described in the ‘Methods’ section) showed that ultrasound-induced damage in the epithelial layers at frequency of 400 kHz was four times higher (*p* < 0.01) *in vivo* (*n* = 6) and 1.8 times higher (*p* < 0.01) *in vitro* (*n* = 12) as compared to respective *in vivo* and *in vitro* sham-treated samples (*n* = 8–33). Using the same method, the damage in the epithelial layer after application of 600-kHz ultrasound was three times higher (*p* < 0.05) *in vivo* (*n* = 6), and two times higher (*p* < 0.01) *in vitro* (*n* = 15) as compared to sham-treated samples (*n* = 8–33).

The *in vitro* corneas [26] are likely to have sustained more tissue damage than the *in vivo* corneas since the *in vitro* corneas were taken out the organism and handled. During *in vivo* study, the eyes were dissected after 90 min from the treatment and were considered as minimally damaged. However, during *in vitro* study, the eyes were shipped from a facility, which dissected the eyes approximately 20–24 h before we received them. There is a chance that these eyes were deteriorated while in the medium and during shipment. This factor may influence the differences between results *in vitro* and *in vivo.* By comparing *in vivo* and *in vitro* sham-treated corneas to the *in vivo* and *in vitro* corneas exposed to 400- and 600-kHz ultrasound, it can be determined whether the tissue damage sustained by the experimental corneas is mainly due to the ultrasound exposure. The *in vivo* corneas usually had mostly intact epithelium, stroma, and endothelium in each of the samples, while all the *in vitro* corneas had large gaps in the epithelium and stroma and had fragmented or absent endothelium. Some of the *in vivo* corneas had more obvious tissue damage which was unlikely due to the ultrasound application. Some of the damages indicated above can be due to histological processing artifacts [30,31]. Our histological analysis indicated that exposing the corneas to 400-kHz ultrasound affected the tissues of the corneas more significantly than exposure to 600-kHz ultrasound, in both *in vitro* and *in vivo* cases. Results from semi-quantitative histological analysis for *in vitro* study showed that approximately two times increase (*p* < 0.01) in the epithelial damage was observed as compared to sham-treated samples (*n* = 33) using 400 kHz (*n* = 12) and 600 kHz (*n* = 15) ultrasound application at 0.8 W/cm^2^ for 5 min. Using the same method of analysis for the same parameters *in vivo* showed four times increase (*p* < 0.01) at frequency of 400 kHz (*n* = 6) and three times increase (*p* < 0.05) at frequency of 600 kHz (*n* = 6), as compared to sham-treated samples (*n* = 8). From the statistical results of layer thickness measurements, we learned that for 400-kHz ultrasound application *in vivo*, the epithelium was significantly thinner (*p* < 0.001) as compared to control cases. The thickness changes in the stroma due to ultrasound application were not statistically significant. The thickness changes in the endothelium could not be analyzed with certainty since the endothelium was completely absent in some samples (and thus recorded as a value of 0), and this absence may have been due to histology processing artifacts as mentioned above.

As an ultrasound wave propagates through a medium, absorption and scattering cause the attenuation of ultrasound energy; transformation of ultrasound energy to heat is a result of this absorption [31,51]. The importance of thermal effects increases with an increase in frequency [34] and is also directly proportional to the ultrasound intensity and duty cycle [31]. Skin permeability and its diffusion coefficient were shown to be increased due to the increase of skin temperature resulting from ultrasound application [32]. In our current *in vivo* study, thermal effects of ultrasound leading to temperature increase in the cornea may have contributed to the increase in the corneal permeability. In this study, the maximum change in temperature was between 0°C and 6°C, with average change of 3.2°C ± 1.8°C for all treatment cases. The corneal permeability to water was shown previously to increase as temperature increased from 24°C to 34°C [52].

Excessive ultrasound energy can thermally and mechanically damage the eye; therefore, U.S. Food and Drug Administration (FDA) has enforced strict thermal and mechanical index limits for ophthalmic applications [53]. FDA guidelines permitted using only *I*_SPTA.3_ of 17 mW/cm^2^ of acoustic energy for ocular application before Medical Device Act of 1976 was passed, which was later changed to *I*_SPTA.3_ of 50 mW/cm^2^[54,55]. Thermal index (TI) is ratio of device's total acoustic power to the power needed to increase tissue's temperature by 1°C [53], and thermal index limit for ocular application is TI < 1.0 [55-57]. We believe that FDA regulations, in case of limits for intensities and temperature increase, are very restricted which may cause a great constraint on using the ultrasound in ocular drug delivery. More investigation using modeling and long-term survival studies is required to determine all potential adverse effects of ultrasound at our parameters in the eye.

The eye is vulnerable to thermal damage from ultrasound exposure because the absence of blood flow in the cornea and lens, both of which are avascular, results in slower cooling as the heat is absorbed by these tissues during ultrasound application [58,59]. Over-heating of the lens is a major concern because it may cause cataract formation; however, Guy et al. indicated that the potential risk would be generated if the temperature increases above 41°C [60]. Measuring the temperature of the lens was not feasible in our current study; therefore, further modeling and experimental investigations are needed to observe the impact of ultrasound application at our proposed parameters on potential heat generation in the lens.

One of the main goals for this study is to investigate the effectiveness of using ultrasound in ocular drug delivery and get some preliminary information regarding safety of ultrasound application. Indeed, future studies will focus on long-term survival experiments (up to 14 days) to determine adverse effects of ultrasound in different eye tissues. Further, lens can get overheated and be damaged as a result of thermal effects of ultrasound application. The hyperthermia level of temperature increase (41°C–43°C) did not reach in our study. The maximum temperature increase during ultrasound application was 31°C, which was below the hyperthermia level; therefore, we believed that the lens was not affected as a result of ultrasound application. The maximal corneal temperature in our experiments (31°C) was below the normal physiological temperature of rabbit cornea of 34°C [61] due to the fact that the exposure to drug solution cooled down the cornea. The maximal corneal temperatures observed in this study were lower than the hyperthermia levels (41°C–43°C); therefore, functional changes in the corneal epithelial cells due to heat were unlikely [58,62]. Modeling studies (that are currently ongoing) can be helpful addressing the changes in the lens, retina, and different tissues in the eye as a result of temperature increase during ultrasound application.

One of the key objectives of this study was to investigate the damages in the cornea due to ultrasound application. Putting a thermocouple inside the cornea would lead to errors in measurement of cornea permeability, which was another goal of this study, and would cause corneal damage, which interferes with the purpose of histology analysis of corneal damages. However, measuring temperature inside the cornea may be more accurate. A modeling study was completed that investigated the issue of temperature increase in different eye tissues.

*In vivo* study was designed to investigate the drug delivery inside the eye while it is intact and inside the body. Also dissecting the eye right after the experiment keeps it fresh which decreases the chance of deterioration. The greater intraocular pressure causes decrease in scleral and corneal permeability. Rudnick et al. indicated that the scleral permeability to small compounds is a weak function of intraocular pressure and mostly depends on molecular weight [63]. However, we did not measure the intraocular pressure and its effect on drug penetration through the cornea. Several animal studies indicated that using HIFU could be a possible and effective technique to reduce intraocular pressure [3]. Ultrasound has the potential for intraocular pressure reduction, which in our case can have an effect on increasing ocular drug permeability; however, this effect in not expected to be major based on previous studies.

The same anesthesia method was performed in both ultrasound treatment and sham treatment cases, so its effects should not impact the study outcome that ultrasound application leads to increase the drug delivery through the cornea. Usually, the depressants of the central nervous system using general anesthetics will decrease intraocular pressure in relation with the depth of anesthesia, and the type of drug can also be a factor effecting the change in intraocular pressure. However, ketamine causes rising on intraocular pressure that is less than the decrease caused by other general anesthesia [64]. There is a possibility that anesthesia can affect drug penetration through the cornea, but this was not investigated in our studies. The sham treatment underwent the same anesthesia methods as ultrasound treatment, which allowed us to observe the effects of ultrasound specifically.

The topical administration of a drug through the cornea, especially for hydrophilic drugs, is a challenging task. Less than 5% of the applied drug can penetrate through the cornea because of the eye barriers [9,11,12]; for example, the epithelium layer has lipophilic properties, which makes it hard for the hydrophilic drug to penetrate into the eye. In other words, we can state that both points made here are important. Enhancement in the delivery of dexamethasone sodium phosphate by 2–3 times is considered clinically significant because this drug has hydrophilic properties and its penetration through the cornea is a challenge.

Even though the results from our *in vivo* study did not show the temperature increase at the hyperthermia level (41°C–43°C), a modeling study (currently in progress) was designed to investigate the temperature changes in the lens, retina, and different tissues in the eye. The results from this modeling study (unpublished data) indicate that the potential temperature increase was minimal and lower than the hyperthermia level in all the eye tissues using ultrasound application at parameters used in *in vivo* study.

## Conclusions

Ultrasound may be used as an effective tool to enhance ocular drug delivery. This study explored the feasibility of using ultrasound for delivery of a clinically relevant drug, dexamethasone sodium phosphate, in an *in vivo* rabbit model. This study was designed using parameters based on the results from our previously performed *in vitro* studies. Increase of 2.8 times (*p* < 0.05) and 2.4 times (*p* < 0.05) in the drug penetration through the cornea was observed using ultrasound at 400 and 600 kHz, respectively. Minor damage and structural changes were present in the corneal epithelium of the ultrasound-treated corneas. Further studies are needed to fully investigate the safety aspects of ultrasound application in ocular drug delivery such as long-term monitoring of recovery of corneal barrier properties and safety of ultrasound exposure in different eye tissues including the lens and retina. It would also be of potential clinical interest to perform studies involving ultrasound application for the enhancement of delivery of anti-fungal and anti-viral ocular drugs currently used in treatment of different eye diseases such as keratitis, scleritis, and herpetic eye disease.

## Competing interests

The authors declare that they have no competing interests.

## Authors’ contributions

MN designed the experimental setup, acquired data, performed all acoustic measurements, analyzed histology slides, and drafted/edited the manuscript with a great help from Dr. VZ. AS helped with analyzing histology slides and statistical analysis and edited the manuscript. SC designed some parts of the eye cup, helped with data collection and animal handling during the experiment, and edited the manuscript. Drs. SM, JL, and CG are our collaborators from GWU Ophthalmology Department and provided knowledge and medical expertise about ophthalmology and ensuring safety of the eye. Dr. VZ supervised MN, AS, and SC in experimental research, monitored all parts of the project, initiated the study, provided the concept and design, and revised/edited the manuscript. All authors read and approved the final manuscript.
